# Calcinosis of the internal jugular vein: Interesting presentation of tertiary hyperparathyroidism

**DOI:** 10.1002/ccr3.6142

**Published:** 2022-08-08

**Authors:** Courtney B. Shires, Mona Shete

**Affiliations:** ^1^ West Cancer Center Germantown Tennessee USA; ^2^ Shete ENT Memphis Tennessee USA

**Keywords:** calcinosis, hyperparathyroidism, internal jugular vein, tracheostomy

## Abstract

Tumoral calcinosis is a severe complication of hemodialysis. A 49‐year‐old male on dialysis for end‐stage renal disease developed a large calcified retropharyngeal mass. This caused stridor and dyspnea, necessitating an emergency awake tracheostomy. This is the first report of internal jugular vein calcinosis. Surgery is recommended.

## Graphical abstract

Tumoral calcinosis with calcification of soft tissues is reported in dialysis patients. Changing from peritoneal dialysis to hemodialysis, discontinuing Vitamin D analogue therapy, changing sevelamer carbonate to lanthan(III)‐carbonate, following a low‐calcium/low‐phosphate diet, and surgery can be treatment options. Parathyroidectomy or excision of calcified tissue are surgical options.

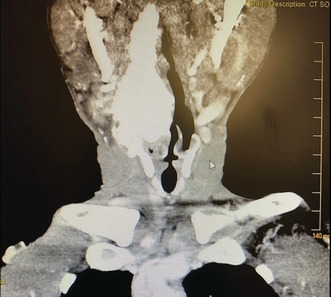

## 1 BACKGROUND

Tumoral calcinosis is a benign rare disease in end stage renal disease (ESRD) patients and is associated with hypercalcemia, hyperphosphatemia, and hyperparathyroidism. This leads to widespread calcifications of soft tissues mostly in the periarticular regions of the large joints.^1^ The pathophysiology of tumoral calcinosis remains mostly unclear. ESRD patients often experience dysregulation of calcium phosphate metabolism due to impaired renal phosphate excretion and vitamin D activation causing hyperparathyroidism, elevated calcium phosphate product, and the subsequent precipitation in soft tissues.^2^ There are multiple case reports of tumoral calcinosis following dialysis, with deposition throughout the hands and hips,^3^ joints,^4^ shoulders and fingertips,^5^ but never specifically in the internal jugular vein (IJV). This is the first case report we are aware of to this point.

## 2 CASE PRESENTATION

**FIGURE 1:**
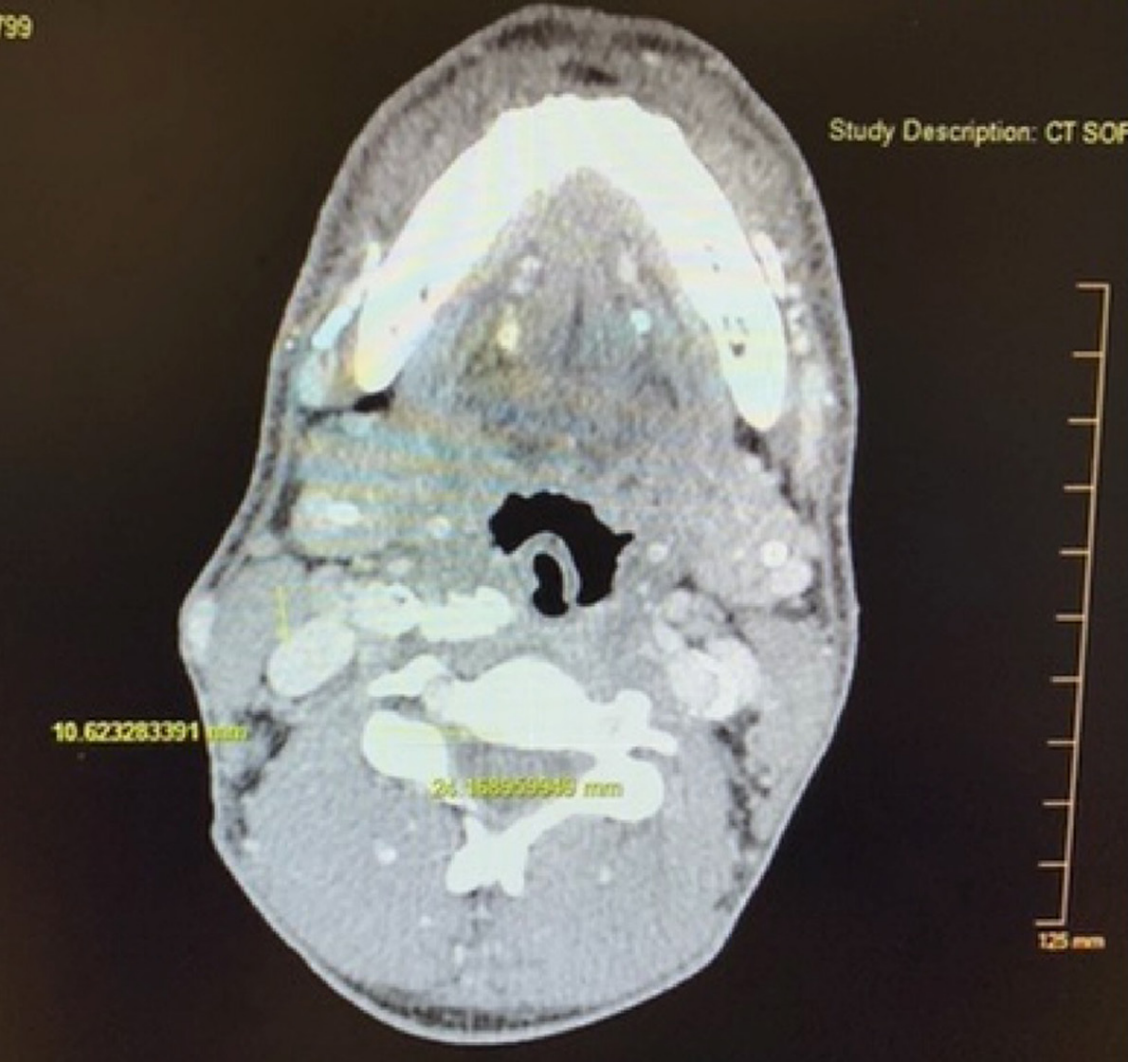
Axial view, CT soft tissue neck. A large calcified lesion in the retropharyngeal area on the right side directly abutting the internal carotid artery

A 49‐year‐old male with a history of dialysis due to ESRD reported pain in his left molars in early November of 2017. He was treated with antibiotics by the health department and was referred to a dentist. The pain then shifted to the right‐side molars. The patient also developed a sore throat on the right side. He then presented to a large municipal hospital on November 4, 2017, denying pain in his teeth but significant pain in his right jaw and throat. He denied fever and difficulty breathing at that time. Computerized tomography (CT) was performed, revealing a large calcified lesion in the retropharyngeal area on the right side directly abutting the internal carotid artery (Figure 1). There was no evidence of dental abscess, retropharyngeal abscess, peritonsillar phlegmon, or fluid collection. Conservative observation was recommended.

**FIGURE 2:**
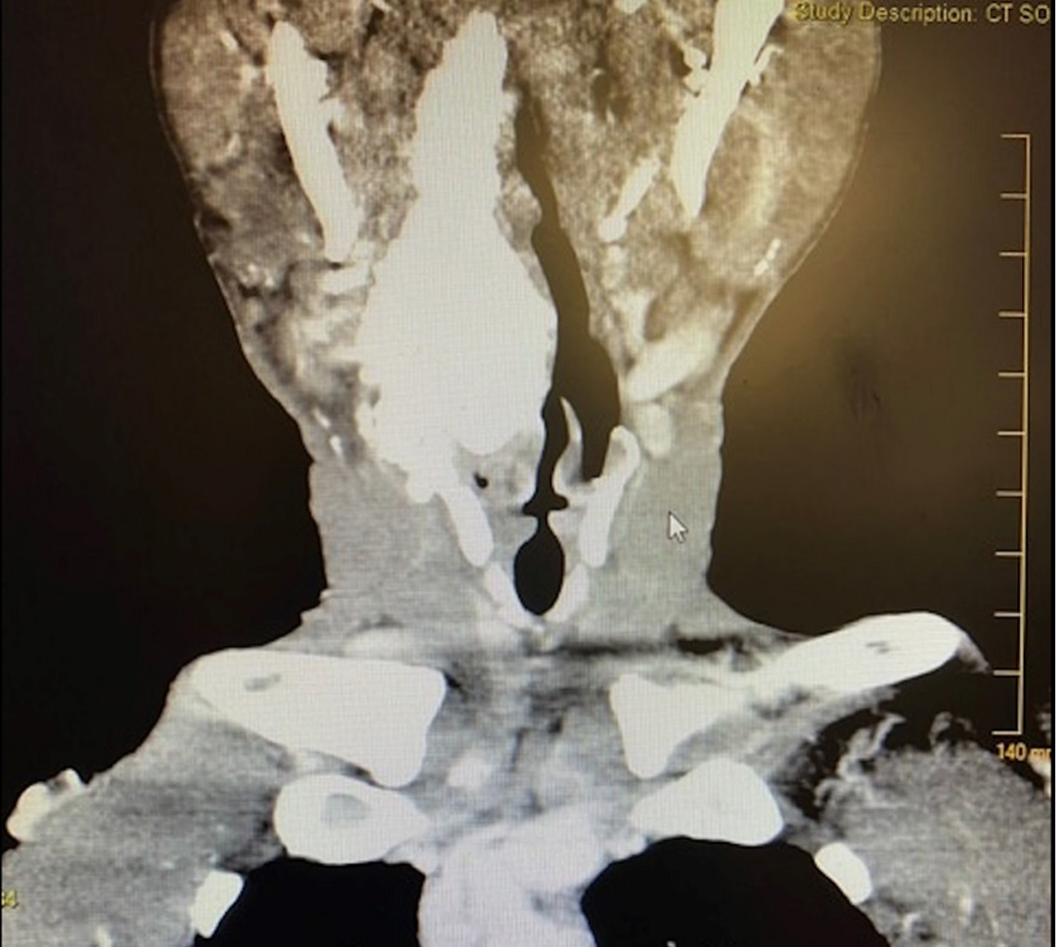
Coronal view, CT soft tissue neck. An amorphous calcified soft tissue mass lesion

**FIGURE 3:**
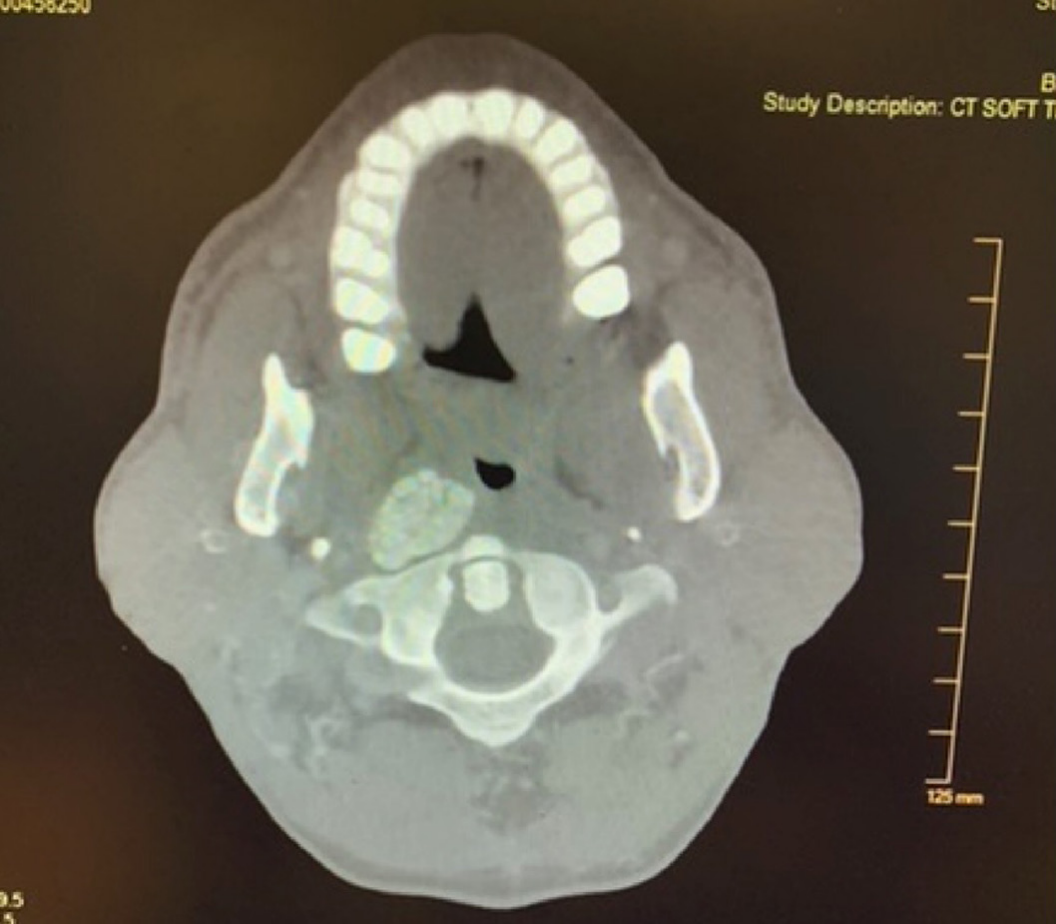
Axial view, CT soft tissue neck. An amorphous calcified soft tissue mass lesion

**FIGURE 4:**
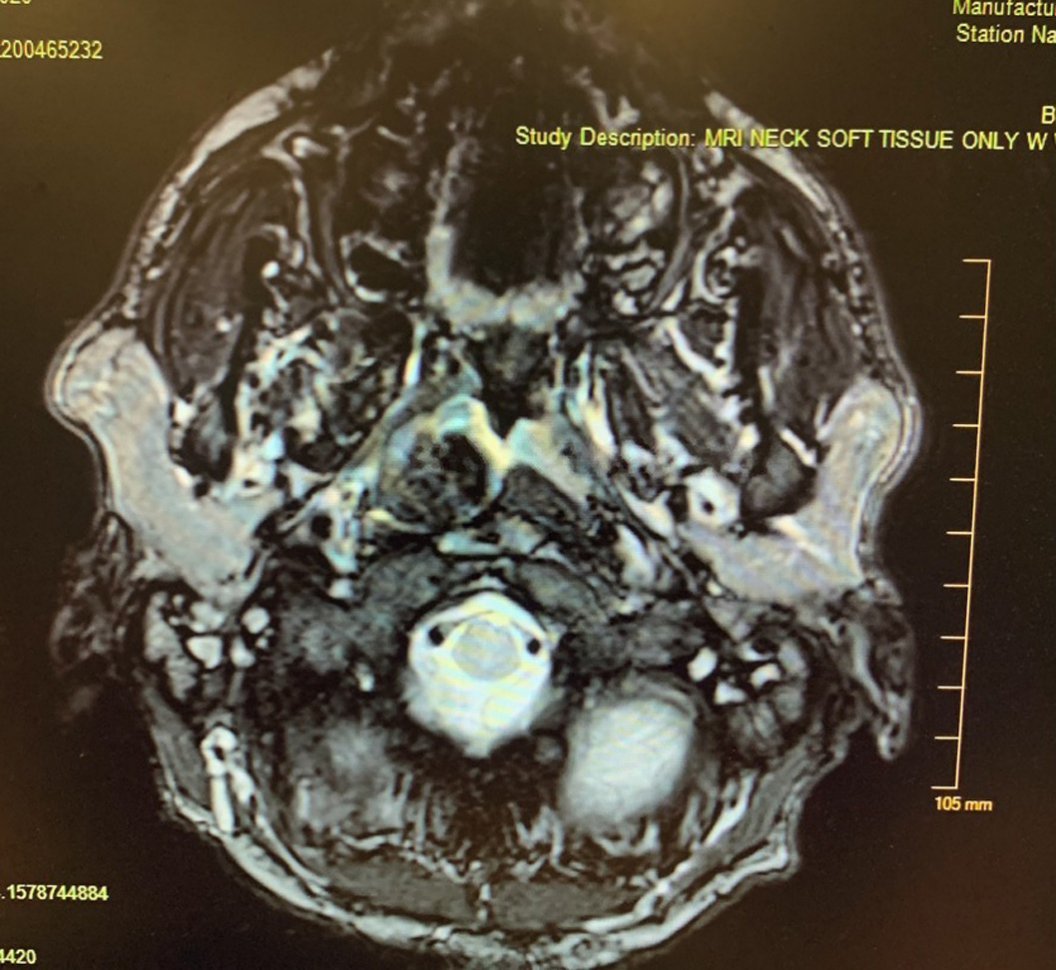
MRI soft tissue neck. Large, right‐sided parapharyngeal mass

**FIGURE 5:**
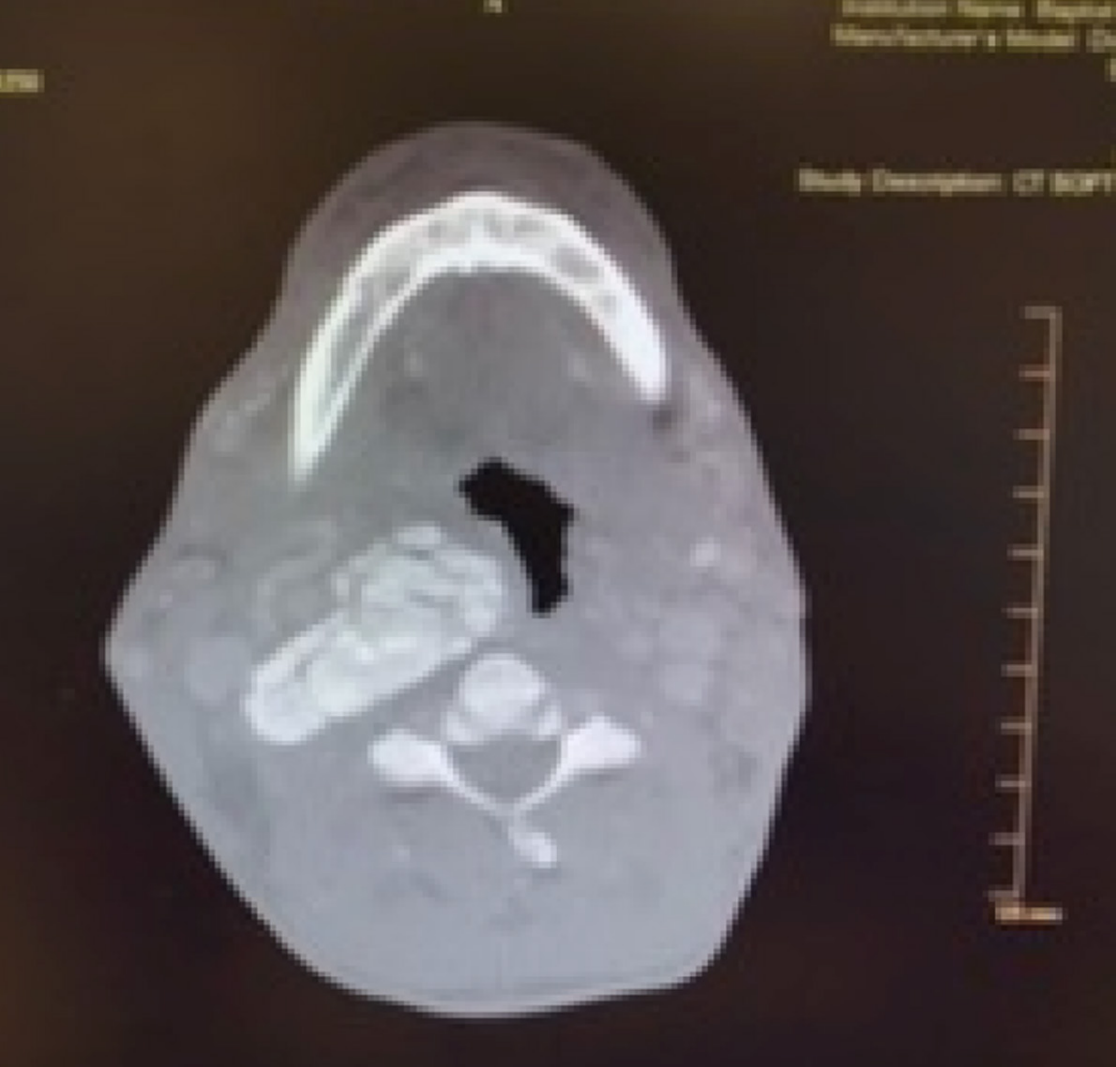
Axial view, CT soft tissue neck. Large multilobulated calcified mass

The patient did not present again until May of 2020. He complained of stridor upon presentation. CT of the chest with contrast was performed on May 11, 2020, showing mild pulmonary edema. CT of the neck with contrast was also performed that day, showing an amorphous calcific soft tissue deposition mass lesion extending from the right posterior pharyngeal aspect to the thyroid lamina with saber‐like narrowing of the upper hypopharyngeal airway (Figures 2 and 3). The size of the calcified mass was noted to be significantly increased relative to what was shown in 2017. Given the appearance on CT of a densely calcified or ossified mass, favored etiologies included metabolic causes for calcification and idiopathic tumoral calcinosis. An x‐ray‐modified barium swallow test was performed on May 12, showing aspiration. A magnetic resonance imaging (MRI) of the neck soft tissue only without contrast was also performed, showing a large, right‐sided parapharyngeal mass extending laterally into the right‐sided deep cervical soft tissues (Figure 4), corresponding with the large multilobulated calcified mass noted on recent CT (Figure 5).

The patient was admitted for stridor and increased difficulty with breathing. He was then transferred to the intensive care unit following hypercapnia and a CO2 narcosis. CBCs and BMPs were drawn and trended, with adjustments made to improve an initial parathyroid hormone of 589.1. Calcium levels were between 9.2 and 10.2, while phosphorous levels remained normal throughout. Medicine, Otolaryngology, and Cardiothoracic Surgery were following the patient on a daily basis, until May 23, 2020, when an emergency tracheostomy had to be performed due to a compromised airway.

He had not been compliant with hemodialysis as an outpatient. As an inpatient, he was maintained on a strict hemodialysis regimen. His selvelamer carbonate was discontinued. He was weaned from the ventilator after his tracheostomy. His calcium and phosphate levels were improving. However, he was then found to have heart failure. He subsequently had cardiac arrest and passed away before any surgery including parathyroidectomy or internal jugular vein sacrifice could be performed.

## 3 DISCUSSION AND CONCLUSIONS

Local trauma, chronic inflammation, and vascular hypoxia have been proposed as potential pathomechanisms for tumoral calcinosis, but a definitive etiology has been evasive.^6^

Adjustments can be made when calcinosis is identified. A patient may change from continuous ambulatory peritoneal dialysis to daily hemodialysis (HD). Vitamin D analogue therapy can be discontinued. Sevelamer carbonate can be discontinued and lanthan(III)‐carbonate used instead. Low‐calcium and low‐phosphate diet should be initiated. The HD schedule can also be changed, for example, from 5 h daytime sessions to 7 h night sessions. Parathyroidectomy can be performed. Follow‐up imaging can then be used to investigate for remission of calcinosis.^7^

Several therapies have been tried for calcinosis with variable results, but surgical excision of calcium deposits remains the primary method of treatment.^6^ For our patient, there was no history of trauma. However, we believe his history of dialysis was the underlying mechanism behind his calcinosis. Previous studies have also shown that reduced patient compliance regarding dialysis treatment contributes to the aggravation of calcinosis in ESRD.^7^, ^8^ In turn, we postulate that the development of calcinosis in our patient was due to poor dialysis compliance. We have not seen a reported case of calcinosis in the IJV, especially with no other associated site of disease systemically.

## AUTHOR CONTRIBUTIONS

Courtney B. Shires, MD, FACS: Collected data, wrote and edited article. Mona Shete, MD: Collected data, wrote and edited article.

## CONFLICT OF INTEREST

The authors report no relevant financial disclosures related to this current work.

## ETHICAL APPROVAL

All issues related to ethics were taken into consideration throughout the study design and proposal and implemented during the research study itself. Informed consent was obtained, beneficence was made a top priority, and respect for confidentiality and privacy were upheld during the study and its various analysis and information assertation components.

## CONSENT

Written informed consent was obtained from the patient to publish this report in accordance with the journal's patient consent policy.

## Data Availability

Other desired data and material relevant to our study is available upon request.
